# The Central Texas Medical Association Meeting

**Published:** 1887-01

**Authors:** 


### Society Notes.

### THE CENTRAL TEXAS MEDICAL ASSOCIATION.

The Central Texas Medical Association met at 10 o'clock Tuesday, Jan. 11th according to adjournment, President F. M. Pitts in the chair, and Secretary H. L. Taylor present. Roll call disclosed a quorum present. The minutes of the previous meeting were read and approved. The president called for the report of the committee on credentials, named at the meeting, October, 1886. The committee appeared to be absent, as a whole. The president appointed Dr. O. I. Halbert, Dr. A. M. Curtis and Dr. R. G. Williams, a new committee on credentials. The committee reported favorably on applications of:

Dr. R. T. Scott, of Perry.
Dr. Albert Horne, of Marlin.
Dr. J. C. Shaw, of Stranger.
Dr. H. M. Stewart, of Patrick.
Dr. Ben. C. Morgan, af Morgan School house.

For the committee on arrangements, its chairman, Dr. H. W. Brown reported that the committee had failed to secure a hall for a permanent place of holding the Association's meetings. The committee was discharged from further duty in that respect.

Dr. F. M. Pitts, president, then delivered an able address, in which he recommended a revision of the constitution and by-laws; and also the formation of a library, museum, laboratory, and the publication of a medical journal, pointing out the advantages of each.

The address received the undivided attention of the Association. At its conclusion Dr. J. C. J. King moved a vote of thanks to the president, which passed unanimously.

### OTHER BUSINESS.

On motion of Dr. J. H. Sears the initiation fee was reduced from $5.00 to $1.00 and the quarterly dues were ordered to remain at $1.00 per quarter, as formerly. It was also ordered that at future meetings seven shall constitute a quorum. The resolutions of Dr. Sears were placed in the hands of the committee on constitution and by-laws in order that the alterations may be made. On motion of Dr. Sears, a committee was appointed to consider the suggestions contained in the address of President Pitts. The committee appointed consisted of Dr. H. W. Brown, Dr. J. C. J. King, Dr. H. L. Taylor and Dr. A. M. Curtis. On motion of Dr. Halbert it was ordered that amounts over $1.00 paid hitherto, as initiation fees, be passed to account of quarterly dues until exhausted. Adjourned for dinner.

AFTERNOON SESSION.

The association met at 3 p. m. The committee on credentials reported favorably on.

Dr. A. M. Douglass, of Oceola.

Dr. R. R. Weir, of Itaska.

Dr. W. B. Carpenter, of Mart.

At the October meeting, Dr. R. P. Tally was appointed to prepare and read a paper as the first subject at the present meeting. He did not report. Dr. W. L. Barker being second, read an interesting outline of an able paper on "Typho Malarial Fever—so-called." It was received into the possession of the association, as its property, and filed with its archives. Much discussion followed the reading by Dr. Barker. Committee on credentials reported favorably on.

Dr. A. H. Snead, of Waco.

Dr. A. M. Armstrong, of Crawford.

Adjourned for supper.

AFTER TEA.

When the association reassembled, Dr. H. L. Taylor, secretary, presented a paper sent by Dr. H. W. Dudley, of Hillsboro, on "Typho-Malarial Fever—so-called." Upon motion the secretary read Dr. Dudley's paper, and it was received and filed. The chair appointed Drs. Douglass, Snead and Armstrong a committee to select subjects of discussion for the next meeting.

Dr. H. L. Taylor read the report of the committee on the president's address. The report was adopted. It reads as follows:

"We, the committee appointed to report on the president's address, beg leave to present the following: A committee of three be appointed on constitution and by-laws, whose duty it shall be to revise the same. That a committee of three be appointed on library and museum, whose duty it shall be to solcit specimens for the museum; books, periodicals and donations for the equipment of the library. That a committee of three be appointed on necrology." The committee asked for further time to report on other portions of the address. The report was adopted. The chair appointed Drs. Brown, Parsons and Howard a committee on constitution and by-laws, with authority to revise the same, and to make additions thereto. Drs. Douglas, Horne and Ghent were appointed on the library and museum committee. Drs. Snead, King and Shaw were appointed a committee on subjects for discussion at the next meeting, reported as follows:

Your committee appointed for the purpose or selecting subjects for the consideration of the association at its next regular meeting, beg leave to report "Puerperal Eclampsia" first, and "Dysentery" second, as the subjects.

After running talk the association adjourned to meet again in this city, the second Tuesday in April

OMITTED.

It is omitted in the above report, to state, that Dr. Snead is appointed on "Puerperal Eclampsia" and Dr. Douglas, on dysentery, to read papers at the next meeting.

